# A Group Affine-Based Inverse Alignment Method for High-Precision Rotational Inertial Navigation Systems

**DOI:** 10.3390/s25061767

**Published:** 2025-03-12

**Authors:** Chao Liu, Ding Li, Huiping Li, Tian Lan, Qixin Lou, Guo Wei, Chunfeng Gao, Ming Tian, Zhongqi Tan, Xudong Yu

**Affiliations:** 1 College of Advanced Interdisciplinary Studies, National University of Defense Technology, Changsha 410073, China; liuchao0622@126.com (C.L.); liding97@nudt.edu.cn (D.L.); 13938115926@163.com (H.L.); a1476073873@163.com (T.L.); louqix@163.com (Q.L.); nudtweiguo@163.com (G.W.); neil1989@126.com (C.G.); tming13@nudt.edu.cn (M.T.); zhqitan@sina.com (Z.T.); 2 Nanhu Laser Laboratory, National University of Defense Technology, Changsha 410073, China

**Keywords:** group affine, initial alignment, inertial navigation system (INS), Lie groups, inverse navigation, rotational INS

## Abstract

Initial alignment plays a pivotal role in inertial navigation systems, as even small orientation errors introduced at startup can significantly degrade subsequent positioning and attitude estimates. In this context, we propose a novel inverse alignment method for rotational inertial navigation that leverages the group affine property and high-speed computing to accelerate and refine the alignment process. Adopting inverse navigation and Lie group theory, we derive a left-invariant error model in the geocentric geosynchronous coordinate framework and rapidly achieve alignment by integrating forward and inverse Kalman filtering. During 2.5-h in-vehicle tests, our approach reduced both the maximum error and CEP (Circular Error Probable 50%) by 60% compared to standard alignment methods, and it surpassed the performance of conventional group affine alignment by improving accuracy by 7.2% and 20%, respectively. These results highlight the method’s ability to deliver swift, precise alignment across diverse initial misalignment angles, offering significant benefits for modern high-precision inertial navigation applications.

## 1. Introduction

Inertial navigation is widely recognized as one of the most fundamental navigation methods currently available. Its primary distinction from other techniques lies in its autonomy: it relies solely on data provided by onboard inertial sensors to determine position, velocity, and attitude. The core component of an inertial navigation system is the Inertial Measurement Unit (IMU), which comprises gyroscopes and accelerometers that independently measure angular velocity and linear acceleration, respectively. By processing these measurements, the system can derive real-time information about the carrier’s attitude, position, and velocity, enabling uninterrupted navigation [1].

A key challenge of inertial navigation is the accumulation of errors over time. To mitigate this, many designs incorporate integrated navigation solutions that fuse inertial data with other navigational inputs. However, in harsh or specialized environments where external aids may be unreliable, inertial navigation offers notable benefits—including self-reliance and resistance to jamming—ensuring continuous navigation when other methods fail. Despite these advantages, the progressive drift of pure inertial navigation compromises accuracy over extended periods [2]. To address this, the technique of rotational modulation is often adopted. It uses a mechanical device to rotate the IMU, thereby reducing the constant drift of gyroscopes and the zero-bias errors of accelerometers, ultimately enhancing overall navigation and positioning accuracy [3].

In rotationally modulated inertial navigation, initial alignment techniques constitute a key area of research. Two critical parameters—alignment time and alignment accuracy—directly influence the performance of the entire rotary-modulated inertial navigation system. Alignment time determines the system’s rapid-start capability, while the resulting alignment accuracy affects subsequent navigation precision. However, these two factors inherently conflict: extending the alignment time generally improves alignment accuracy and thus enhances navigation performance [4,5]. To achieve superior alignment outcomes and strike an optimal balance between time and accuracy, researchers have explored various approaches. The conventional method features a two-step process: first performing a coarse alignment to obtain approximate attitude data, then using this approximation as the starting point for fine alignment [6].

For coarse alignment, scholars often leverage the geometric relationship between Earth’s rotation and gravity, the apparent movement of gravity, or integrate external velocity sensors (such as odometers or Doppler velocity logs) to derive initial attitude estimates [7,8,9,10,11]. Meanwhile, fine alignment research commonly employs advanced filtering, sensor fusion, or external velocity inputs to achieve higher accuracy under dynamic conditions [12,13,14,15,16,17].

To expedite the initial alignment process, the backtracking algorithm leverages the efficient computational power of computers to record gyroscope and accelerometer data in real time. The iterative application of this data through forward and inverse navigation processes helps reduce the amount of IMU output data required to converge on accurate misalignment angles. For instance, Gao et al. [18] applied this concept to the precise alignment of the Kalman filter under static mooring conditions, developing a forward-looking data processing technique that effectively utilizes stored IMU information to quickly ascertain the initial attitude angle. Li et al. [19] recorded data during the coarse alignment phase and executed the Kalman filter’s precise alignment in inverse from the data’s starting point, innovatively achieving forward backtracking of fine alignment relative to coarse alignment, thereby significantly reducing overall alignment time and enhancing speed. The OBA method based on the backtracking algorithm proposed by Chang et al. [20] incorporates this algorithm into alignment optimization and introduces a backtracking integration technique for constructing vector observations, which fully exploits IMU data and generates more non-collinear observation vectors with a single inverse construction, thus improving the accuracy of the OBA method within a limited alignment timeframe. Lou [21] proposed a closed-loop backtracking coarse alignment method based on the Kalman filter and designed a piecewise integral vector construction approach, enabling the algorithm to construct more non-collinear vectors during multiple backtracking iterations, further reducing the data requirements for coarse alignment and enhancing alignment accuracy.

The traditional alignment method, which consists of coarse alignment followed by fine alignment, has several drawbacks: it requires two steps, exhibits relatively slow convergence speed, takes a long time to complete, and does not achieve high accuracy in the short term. Additionally, when dealing with large misalignment angles, an initial coarse alignment step is required to reduce the misalignment error, and this approach also faces challenges in the allocation of alignment time. To tackle these issues, Li [22] introduced a fast alignment method for rotational inertial navigation using group affine techniques. This approach aims to resolve the problems of alignment time allocation and parameter settings during the initial alignment process of rotational modulation inertial navigation systems, achieving high accuracy in a shorter timeframe. Given that many researchers have noted that the backtracking algorithm can enhance convergence speed and reduce alignment time, we are considering whether this algorithm could be effectively applied to the group affine rotational inertial navigation alignment process.

**Figure 1:**
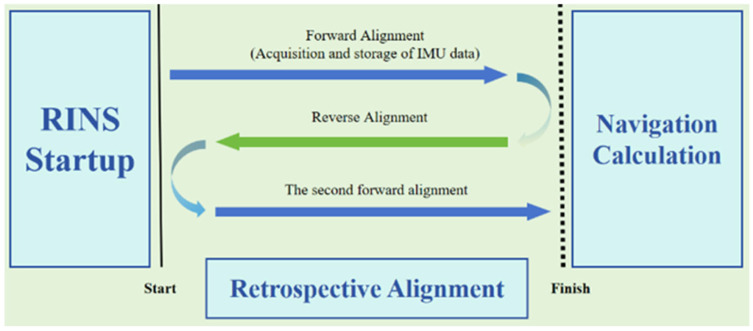
Alignment scheme schematic.

Fortunately, both our theoretical derivations and experimental validations confirm the feasibility of this idea, leading us to introduce a renewed inverse alignment scheme for group affine rotational inertial navigation by integrating the backtracking concept into the initial alignment process originally proposed by Li [22]. This approach significantly enhances the accuracy of the initial alignment without extending its duration, achieving a major breakthrough in finding the optimal balance between alignment time and accuracy. Figure 1 is a schematic diagram of the inverse alignment process.

In this scheme, after the navigation computer has been running for a certain period, it first records and processes the navigation data from start to finish. Simultaneously, the RINS (Rotational Inertial Navigation System) performs a forward alignment operation for the same time period. Then, with the help of the computer’s efficient processing capabilities, the alignment process is inversed from the completion moment back to the start time and is carried out within a short period. Finally, the system realigns from the start time to the finish time to catch up with the real-time navigation progress. This method effectively improves the accuracy of the initial alignment by reusing limited experimental data for multiple times and provides a new perspective for improving the performance of inertial navigation systems.

## 2. Inverse RINS Mechanization and Mathematical Preliminaries

The research content of this paper focuses on the inverse alignment of the RINS, which involves two parts: forward alignment and inverse alignment. Preliminary research on the mechanization and mathematics of the forward RINS has been presented in [22], so this paper will only introduce the research on the mechanization and mathematics of the inverse RINS.

### 2.1. Introduction to Related Coordinate Systems

Geocentric inertial system (i-frame): the origin $o_{i}$ is the Earth’s center of mass, the $o_{i}x_{i}$ axis points to the equinox in the equatorial plane, the $o_{i}y_{i}$ axis is perpendicular to the $o_{i}x_{i}$ axis in the equatorial plane, and the $o_{i}z_{i}$ axis is the Earth’s rotation axis.

Navigation coordinate system (n-frame): the origin $o_{n}$ is the center of the carrier, the $o_{n}x_{n}$ axis points geographically east, the $o_{n}y_{n}$ axis points geographically north, and the $o_{n}z_{n}$ axis points skyward.

Earth coordinate system (e-frame): The origin $o_{e}$ is the center of the Earth, $o_{e}x_{e}$ and $o_{e}y_{e}$ axes are in the plane of the Earth’s equator, where $o_{e}x_{e}$ points to the prime meridian, and the $o_{e}z_{e}$ axis is the axis of the Earth’s rotation and points to the North Pole. The e-system is solidly connected to the Earth and is also referred to as the Geocentric Earth Solid Coordinate System (Earth-Centered Earth-Fixed, ECEF).

Carrier coordinate system (b-frame): the center of mass of the carrier is the origin $o_{b}$, the $o_{b}x_{b}$ axis points to the right along the horizontal axis from the origin, the $o_{b}y_{b}$ axis points to the front along the vertical axis from the origin, and the $o_{b}z_{b}$ axis is perpendicular to the horizontal plane of the carrier and points to the top from the origin.

IMU sensor coordinate system (s-frame): $o_{s}$ is the origin and is the intersection of the various sensitive axes, the $o_{s}x_{s}$, $o_{s}y_{s}$, and $o_{s}z_{s}$ oz axes correspond to the sensitivity axes of the three gyroscopes and accelerometers, respectively.

### 2.2. Inverse Navigation Algorithms

The attitude, velocity, and position update equations for the strapdown RINS can generally be expressed as [1]

(1) $$Q_{sk}^{n}=Q_{n(k-1)}^{n(k)}Q_{sk-1}^{n}Q_{s(k)}^{s(k-1)}$$

(2) $$v_{k}^{n}=v_{k-1}^{n}+T_{s}\left[C_{sk-1}^{n}\right.f_{k}^{s}-\left(2\omega_{iek-1}^{n}+\omega_{enk-1}^{n}\right)\times v_{k-1}^{n}+\left.g^{n}\right]$$

(3) $$L_{k}=L_{k-1}+T_{s}v_{Nk-1}^{n}/(R_{M}+h_{k-1})$$

(4) $$\lambda_{k}=\lambda_{k-1}+T_{s}v_{Ek-1}^{n}\sec L_{k-1}/(R_{N}+h_{k-1})$$

(5) $$h_{k}=h_{k-1}+T_{s}v_{Uk-1}^{n}$$

(6) $$\mathrm{where}\;\omega_{enk}^{n}=\left[-\frac{v_{Nk}^{n}}{R_{M}+h_{k}}\;\frac{v_{Ek}^{n}}{R_{N}+h_{k}}\;\frac{v_{Ek}^{n}\tan L_{k}}{R_{N}+h_{k}}\right]$$

(7) $$\omega_{iek}^{n}=\left[\begin{array}{c} 0\\ \omega_{ie}\cos L_{k}\\ \omega_{ie}\sin L_{k} \end{array}\right]$$

(8) $$g^{n}=\left[\begin{array}{c} 0\\ 0\\ g \end{array}\right]$$

Derivation of (1)–(5) yields the attitude, position, and velocity updating equations for rotary inertial guidance inverse navigation [23], respectively:

(9) $$Q_{sk-1}^{n}=Q_{n(k)}^{n(k-1)}Q_{sk}^{n}Q_{s(k-1)}^{s(k)}$$

(10) $$v_{k-1}^{n}=v_{k}^{n}+T_{s}\left[C_{sk-1}^{n}(-\right.f_{k}^{s})-(2(-\omega_{iek-1}^{n})+(-\omega_{enk}^{n}))\times v_{k}^{n}+(-\left.g^{n})\right]$$

(11) $$L_{k-1}=L_{k}+\left[-(T_{s}v_{Nk-1}^{n}/(R_{M}+h_{k}))\right]$$

(12) $$\lambda_{k-1}=\lambda_{k}+\left[-(T_{s}v_{Ek-1}^{n}\sec L_{k-1}/(R_{N}+h_{k-1}))\right]$$

(13) $$h_{k-1}=h_{k}+\left[-(T_{s}v_{Uk-1}^{n})\right]$$

$Q_{sk}^{n}$ is the pose quaternion at moment k and $Q_{sk-1}^{n}$ is the pose quaternion at moment k − 1. $Q_{n(k-1)}^{n(k)}$ is the transformed quaternion in the n-system from k − 1 to moment k, which can be derived from the angular velocity of rotation of the Earth $\omega_{ie}^{n}$ and the angular velocity of rotation of the n-system relative to the Earth $\omega_{en}^{n}$. $Q_{s(k)}^{s(k-1)}$ is the transformed quaternion of the s-system from moment k − 1 to moment k, which can be obtained from the angular rate $\omega_{is}^{s}$ of the IMU’s gyroscope output. $v^{n}$ is the rotating inertial navigation system’s velocity in the n-system. L, λ, and h indicate the latitude, longitude, and elevation of the carrier’s location, respectively. $T_{s}$ is the sampling period of the gyroscope and accelerometer and $f^{s}$ is the specific force. $g^{n}$ is the acceleration of gravity in the n-system. $R_{M}$ refers to the radius of curvature of the meridian (the north–south direction). This radius describes the curvature of the Earth’s surface along the lines of longitude. Its value varies with latitude, being larger at the poles and smaller at the equator, and $R_{N}$ refers to the radius of curvature of the parallel (the east–west direction). It represents the curvature of the Earth’s surface along the lines of latitude. Like $R_{M}$, this value also changes with latitude, reaching its maximum at the equator and diminishing as one moves toward the poles.

The derived formula for inverse navigation shows that the realization of inverse navigation requires the inverse operation of the angular rate $\omega_{is}^{s}$ of the gyro output, the specific force $f^{s}$ of the accelerometer output, the gravity acceleration $g^{n}$, the angular velocity of the Earth’s rotation $\omega_{ie}^{n}$, the angular velocity of the n-system’s rotation with respect to the Earth $\omega_{en}^{n}$, and the amount of positional change.

### 2.3. Inverse RINS Mechanization on Lie Group Theory

According to [24], group affine systems possess several unique attributes:

1. State–Trajectory Independence:

This fundamental property emphasizes that the left/right invariant error is independent of the system trajectory, which is a central tenet of the group affine model. This independence ensures that the discrepancy between the true state and the estimated state is unaffected by the actual trajectory of the system. Consequently, it simplifies the design of observers for group affine systems by decoupling the error dynamics from the complexity of the system’s path.

2. Local Linearization Stability:

This characteristic focuses on the stability analysis of linearized models in Euclidean space. It allows for a simplified assessment of the stability of the linearized model, as it is independent of the system’s global state estimates. This simplification is valuable for evaluating the stability properties of group affine systems.

3. Precise Nonlinear Error Inversion in the Lie Group:

This property highlights the accurate transformation from the linearized model to the nonlinear error within the Lie group. It facilitates the efficient computation of the nonlinear error in the context of the Lie group based on the linearized model. This capability is crucial for designing effective observers, as it enables us to leverage the stability analysis of the linearized model for the nonlinear error.

In general, the attitude, velocity, and position differential equations for the RINS in the n-system are [25]

(14) $$\dot{C_{s}^{n}}=C_{s}^{n}\left(\omega_{is}^{s}\times \right)-\left(\omega_{in}^{n}\times \right)C_{s}^{n}$$

(15) $$v_{k-1}^{n}=v_{k}^{n}+T_{s}\left[C_{sk-1}^{n}(-\right.f_{k}^{s})-(2(-\omega_{iek-1}^{n})+(-\omega_{enk}^{n}))\times v_{k}^{n}+(-\left.g^{n})\right]$$

(16) $${\dot{p}}^{n}=R_{c}v^{n}$$

(17) $$\mathrm{where}\;R_{c}=\left[\begin{array}{ccc} 0 & \frac{1}{R_{M}+h} & 0\\ \frac{\sec L}{R_{N}+h} & 0 & 0\\ 0 & 0 & 1 \end{array}\right]$$

(18) $$p^{n}=\left[\begin{array}{c} L\\ \lambda \\ h \end{array}\right]$$

(19) $$v^{n}=\left[\begin{array}{ccc} v_{E}^{n} & v_{N}^{n} & v_{U}^{n} \end{array}\right]$$

(20) $$\omega_{in}^{n}=\omega_{ie}^{n}+\omega_{en}^{n}$$

$C_{s}^{n}$ is the attitude transformation matrix from the s-system to the n-system, $p^{n}$ is the position vector of the IMU in the n-frame.

From (6)–(8) and (14)–(20), it can be seen that when attitude updating is performed it is affected by the velocity $v^{n}$ and latitude L.

In order to construct the error equation of state for our rotating inertial guidance system that satisfies the state–trajectory independence property, decoupling between the state quantities is required. This problem can be solved by transforming the attitude differential equations in the n-system to the e-system. Based on the decoupled forward RINS mechanization of [22], then the inverse navigation algorithm can be used to derive a new attitude differential equation

(21) $$\dot{C_{s}^{e}}=C_{s}^{e}\left(-\omega_{is}^{s}\times \right)-\left(-\omega_{ie}^{e}\times \right)C_{s}^{e}$$

where $C_{s}^{e}$ represents the transformation matrix of the attitude from the IMU coordinate system to the ECEF.

Similarly, the velocity and position differential equations also need to undergo decoupling operation, and the new velocity differential equation obtained is

(22) $${\dot{\bar{v}}}^{e}=C_{s}^{e}\left(-f^{s}\right)-\left(-\omega_{ie}^{e}\times \right){\bar{v}}^{e}+{\bar{g}}^{e}$$

${\bar{v}}^{e}$ represents the auxiliary velocity vector and $v^{e}$ denotes the gravitational field associated with the reference ellipsoid. There is a relationship between the following equations

(23) $${\bar{v}}^{e}=v^{e}+\left(-\omega_{ie}^{e}\times \right)p^{e}$$

(24) $${\bar{g}}^{e}=g^{e}+{\left(-\omega_{ie}^{e}\times \right)}^{2}p^{e}$$

(25) $$g^{e}=C_{n}^{e}\left(-g^{n}\right)$$

$C_{n}^{e}$ is the transformation matrix from the n-system to the e-system.

The new differential equation of position is

(26) $${\dot{p}}^{e}={\bar{v}}^{e}-\left(-\omega_{ie}^{e}\times \right)p^{e}$$

According to (21), (22) and (26), we can derive the state error differential equations which defines in $SO\left(3\right)+{\mathbb{R}}^{6}$, for attitude, velocity and position in the inverse direction as follows:

(27) $$\dot{\varphi }=-\left(-{\tilde{\omega }}_{\mathrm{is}}^{s}\times \right)\varphi -(-\delta \omega_{\mathrm{is}}^{s})$$

(28) $$\delta {\overset{\dot{¯}}{v}}^{e}=-\left(-\tilde{f^{s}}\times \right)\varphi -\left(-{\tilde{\omega }}_{\mathrm{is}}^{s}\times \right)\delta {\bar{v}}^{e}-(-\delta f^{s})$$

(29) $$\delta {\dot{p}}^{e}=\delta {\bar{v}}^{e}-\left(-\omega_{ie}^{e}\times \right)\delta p^{e}$$

where φ, $\delta {\bar{v}}^{e}$, and $\delta p^{e}$ denote the attitude state error, auxiliary velocity state error, and position state error, respectively. ${\tilde{\omega }}_{\mathrm{is}}^{s}$ represents the angular rate of the gyro output with some error $\delta \omega_{is}^{s}$, which can be expressed as

(30) $${\tilde{\omega }}_{\mathrm{is}}^{s}=\omega_{is}^{s}+\delta \omega_{is}^{s}$$

$\tilde{f^{s}}$ is expressed as the specific force of the accelerometer output with some error $\delta f^{s}$, which can be expressed as

(31) $$\tilde{f^{s}}=f^{s}+\delta f^{s}$$

Combine the attitude transition matrix $C_{s}^{e}$, the auxiliary velocity matrix ${\bar{v}}^{e}$, and the position matrix $p^{e}$ derived above into a new matrix named

(32) $$\chi =\left[\begin{array}{ccc} C_{s}^{e} & {\bar{v}}^{e} & p^{e}\\ 0_{1\times 3} & 1 & 0\\ 0_{1\times 3} & 0 & 1 \end{array}\right]\in {SE}_{2}\left(3\right)$$

(33) $$\begin{array}{l} \dot{\chi }=f_{u}\left(\chi \right)\\ =\chi \left[\begin{array}{ccc} \left(\omega_{is}^{s}\times \right) & f^{s} & 0\\ 0 & 0 & 1\\ 0 & 0 & 0 \end{array}\right]+\left[\begin{array}{ccc} -\left(\omega_{ie}^{e}\times \right) & {\bar{g}}^{e} & 0\\ 0 & 0 & -1\\ 0 & 0 & 0 \end{array}\right]\chi \\ =\chi W+U\chi \end{array}$$

According to the derivation of [22], it can be shown that χ is satisfying the group affine property. $\dot{\chi }$ is the derivative of χ.

### 2.4. Left-Invariant Error State Model in Inverse RINS

In this paper, we use a left-invariant error model, the advantages of which have been described in [26,27], and the left-invariant error model used in this paper is briefly described next.

The left-invariant error $\eta_{l}$ can be obtained by combining χ and $\tilde{\chi }$ as follows:

(34) $$\eta_{l}={\tilde{\chi }}^{-1}\chi =\left[\begin{array}{ccc} {\tilde{C}}_{s}^{eT}C_{s}^{e} & {\tilde{C}}_{s}^{eT}\left({\bar{v}}^{e}-\tilde{{\bar{v}}^{e}}\right) & {\tilde{C}}_{s}^{eT}\left(p^{e}-{\tilde{p}}^{e}\right)\\ 0_{1\times 3} & 1 & 0\\ 0_{1\times 3} & 0 & 1 \end{array}\right]$$

where, $\tilde{\chi }$ is an estimate of χ.

From [27] the following equation can be derived:

(35) $$\begin{array}{ll} {\dot{\eta }}_{l} & =g_{u}\left(\eta_{l}\right)=f_{u}\left(\eta_{l}\right)-f_{u}\left(I_{5}\right)\eta_{l}\\ & =\eta_{l}W+U\eta_{l}-\left(W+U\right)\eta_{l}\\ & =\eta_{l}W-W\eta_{l} \end{array}$$

Equation (35) proves that the left-invariant error differential equation in the inverse RINS is trajectory independent [22].

Based on the above proofs of the properties of the left-invariant error model, we can derive the differential equations for the attitude error, velocity error, and position error of the left-invariant error model in the inverse RINS, respectively.

Usually the angle of $\varphi_{l}$ is very small, then ${\tilde{C}}_{s}^{eT}C_{s}^{e}$ can be described in terms of $\varphi_{l}$ as

(36) $$dC_{s}^{e}≜{\tilde{C}}_{s}^{eT}C_{s}^{e}\approx I_{3}+\left(\varphi_{l}\times \right)$$

Use ${\tilde{C}}_{s}^{eT}C_{s}^{e}$ to denote the attitude error $dC_{s}^{e}$ and $\varphi_{l}$ to denote the Euler angle error in the left-invariant error model, corresponding to the attitude error $dC_{s}^{e}$.

The attitude, velocity, and position error vectors of the elements in (32) corresponding to the left-invariant error model can be derived as follows [25]:

(37) $$d{\bar{v}}_{l}^{e}={\tilde{C}}_{s}^{eT}\left({\bar{v}}^{e}-\tilde{{\bar{v}}^{e}}\right)=-{\tilde{C}}_{s}^{eT}\delta {\bar{v}}^{e}$$

(38) $$dp_{l}^{e}={\tilde{C}}_{s}^{eT}\left(p^{e}-{\tilde{p}}^{e}\right)=-{\tilde{C}}_{s}^{eT}\delta p^{e}$$

The new differential equation for the velocity error is

(39) $$d\dot{{\bar{v}}_{l}^{e}}=-{\tilde{C}}_{s}^{eT}\delta {\bar{v}}^{e}-{\dot{\tilde{C}}}_{s}^{eT}\delta v^{e}$$

Further, from Equation (28) it can be derived that

(40) $$d\dot{{\bar{v}}_{l}^{e}}=-\left(-\tilde{f^{s}}\times \right)\varphi_{l}-\left(-{\tilde{\omega }}_{\mathrm{is}}^{s}\times \right)d{\bar{v}}_{l}^{e}-(-\delta f^{b})$$

Similarly, the new attitude and position error differential equations can be derived from (27) and (29), respectively:

(41) $$d{\dot{p}}_{l}^{e}=d{\bar{v}}_{l}^{e}-\left(-{\tilde{\omega }}_{\mathrm{is}}^{s}\times \right)dp_{l}^{e}$$

(42) $${\dot{\varphi }}_{l}=-\left(-{\tilde{\omega }}_{\mathrm{is}}^{s}\times \right)\varphi_{l}-(-\delta \omega_{is}^{s})$$

From (40)–(42), we can obtain the expression for ${\dot{\eta }}_{l}$ as

(43) $$\begin{array}{l} {\dot{\eta }}_{l}=\left[\begin{array}{cc} -\left(-{\tilde{\omega }}_{\mathrm{is}}^{s}\times \right)\varphi_{l}-(-\delta \omega_{is}^{s}) & -\left(-\tilde{f^{s}}\times \right)\varphi_{l}-\left(-{\tilde{\omega }}_{\mathrm{is}}^{s}\times \right)d{\bar{v}}_{lg}^{e}-(-\delta f^{b})\\ 0_{1\times 3} & 0\\ 0_{1\times 3} & 0 \end{array}\right.\begin{array}{c} ...\\ ...\\ ... \end{array}\\ \left.\begin{array}{ccc} & ... & d{\bar{v}}^{e}-\left(-{\tilde{\omega }}_{\mathrm{is}}^{s}\times \right)dp_{lg}^{e}\\ & ... & 0\\ & ... & 0 \end{array}\right] \end{array}$$

The model established in this paper is the model of the ring laser gyroscope inertial navigation system, and the gyroscope error model $\delta \omega_{is}^{s}$ and accelerometer error model $\delta f^{s}$ are generally composed of constant error and random error, which are expressed as follows [22]:

(44) $$\delta \omega_{is}^{s}=\varepsilon^{s}+w_{g}^{s}$$

(45) $$\delta f_{is}^{s}=\nabla^{s}+w_{a}^{s}$$

where $\varepsilon^{s}$ denotes the gyroscope drift error, $\nabla^{s}$ denotes the accelerometer zero-bias error, and $w_{g}^{s}$ and $w_{a}^{s}$ denote the zero-mean Gaussian noise of the gyroscope and accelerometer, respectively.

From the differentiation of the left-invariant error of (43), it can be seen that it is ‘State–Trajectory Independence’ and is consistent with the group affine characterization.

## 3. Inverse RINS Alignment State Model

In this study, we use a two-position alignment scheme, which generally results in better performance of the initial alignment. However, when performing the rotation step of dual-position alignment, the center of the rotating mechanism is not perfectly coincident at the inertial measurement unit (IMU), and a certain distance (i.e., lever arm error) exists, and linear motion disturbances often occur. The existence of the lever arm error causes the observation matrix of the error model to no longer satisfy the nature of the group affine, which can lead to some errors in the output and problems in the alignment results. Therefore, in order to eliminate this effect, we add the lever arm error to the error state equation to ensure that our alignment results are optimal.

### 3.1. Inverse RINS Error State Model

We model the inverse RINS error state as

(46) $$d{\dot{x}}_{lg}=F_{lg}dx_{lg}+G_{lg}w$$

In the previous sections we derived the error equations in the inverse RINS alignment, so the error state vector in the error model is

(47) $$dx_{lg}={\left[\begin{array}{cccccc} \varphi_{l} & d{\bar{v}}_{l}^{e} & dp_{l}^{e} & \varepsilon^{s} & \nabla^{s} & L^{s} \end{array}\right]}^{T}$$

The new state transfer matrix $F_{lg}$ and system noise matrix $G_{lg}$ are shown below [27]:

(48) $$F_{lg}=\left[\begin{array}{cccccc} \left({\tilde{\omega }}_{\mathrm{is}}^{s}\times \right) & 0_{3} & 0_{3} & I_{3} & 0_{3} & 0_{3}\\ \left({\tilde{f}}_{\mathrm{is}}^{s}\times \right) & \left({\tilde{\omega }}_{\mathrm{is}}^{s}\times \right) & 0_{3} & 0_{3} & I_{3} & 0_{3}\\ 0_{3} & I_{3} & \left({\tilde{\omega }}_{\mathrm{is}}^{s}\times \right) & 0_{3} & 0_{3} & 0_{3}\\ 0_{3} & 0_{3} & 0_{3} & 0_{3} & 0_{3} & 0_{3}\\ 0_{3} & 0_{3} & 0_{3} & 0_{3} & 0_{3} & 0_{3} \end{array}\right]$$

(49) $$G_{lg}=\left[\begin{array}{cc} I_{3} & 0_{3}\\ 0_{3} & I_{3}\\ 0_{12\times 3} & 0_{12\times 3} \end{array}\right]$$

### 3.2. Inverse RINS Error Observation Model

According to [27], we can derive the error observation equation for the inverse RINS alignment as follows:

(50) $$\begin{array}{ll} Z_{lg} & ={\tilde{v}}^{e}-\tilde{C_{s}^{e}}v^{s}\\ & =-\left(v^{e}\times \right)C_{s}^{e}\varphi_{l}+\delta v^{e}-\tilde{C_{s}^{e}}v_{L}\\ & =-\left[{\bar{v}}^{e}-\left(-\omega_{ie}^{e}\times \right)p^{e}\right]\times C_{s}^{e}\varphi_{l}+\delta {\bar{v}}^{e}-...\\ & \left(-\omega_{ie}^{e}\times \right)\delta p^{e}-\tilde{C_{s}^{e}}\left(-\omega_{is}^{s}\times L^{s}\right)\\ & =-\left[{\bar{v}}^{e}-\left(-\omega_{ie}^{e}\times \right)p^{e}\right]\times C_{s}^{e}\varphi_{l}-\tilde{C_{s}^{e}}d{\bar{v}}^{e}+...\\ & \left(-\omega_{ie}^{e}\times \right)\tilde{C_{s}^{e}}dp^{e}-\tilde{C_{s}^{e}}\left(-\omega_{is}^{s}\times L^{s}\right) \end{array}$$

From the derivation results, we can see that $\left(-\omega_{is}^{s}\times L^{s}\right)$ is the linear motion disturbance caused by the lever arm error $L^{s}$ that we mentioned earlier, and we can prove that the equation we constructed is accurate.

According to the error observation Equation (50), we can build the observation matrix $H_{lg}$ as

(51) $$\begin{array}{c} H_{lg}=\left[\begin{array}{cccc} \left[-\left[\left({\bar{v}}^{e}\times \right)+\left(-\omega_{ie}^{e}\times p^{e}\right)\times \right]\right]C_{s}^{e} & -\tilde{C_{s}^{e}} & \left(-\omega_{ie}^{e}\times \right)\tilde{C_{s}^{e}} & ... \end{array}\right.\\ \begin{array}{ccc} & & \left.\begin{array}{ccc} ... & 0_{3\times 6} & \tilde{C_{s}^{e}}\left(-\omega_{is}^{s}\times \right) \end{array}\right] \end{array} \end{array}$$

According to (51), we can see that it is contradictory for the observation matrix to be related to the global variables ${\bar{v}}^{e}$, $p^{e}$, and $\tilde{C_{s}^{e}}$ and to be independent of the global state variables of the group affine nature. In engineering applications, the ground velocity of a two-position RINS during initial alignment is usually 0, and $v^{e}=0$ can be assumed. We can derive the following:

(52) $${\bar{v}}^{e}-\left(-\omega_{ie}^{e}\times \right)p^{e}=0$$

Substituting (52) into (51), the observation matrix is given by the following formula:

(53) $$H_{lg}=\left[\begin{array}{ccccc} 0_{3\times 3} & -\tilde{C_{s}^{e}} & \left(-\omega_{ie}^{e}\times \right)\tilde{C_{s}^{e}} & 0_{3\times 6} & \tilde{C_{s}^{e}}\left(-\omega_{is}^{s}\times \right) \end{array}\right]$$

It can be seen from (53) that the observation matrix $H_{lg}$ that we constructed has no connection to the global variables ${\bar{v}}^{e}$ and $p^{e}$, but it is still related to the global variable $\tilde{C_{s}^{e}}$. It is known from [24,26] that we can make the following transformation:

(54) $${\bar{Z}}_{lg}={\tilde{C}}_{s}^{eT}Z_{lg}$$

The new observation matrix is obtained as follows:

(55) $${\bar{H}}_{lg}={\tilde{C}}_{s}^{eT}H_{lg}=\left[\begin{array}{ccccc} 0_{3\times 3} & -I_{3} & {\tilde{C}}_{s}^{eT}\left(-\omega_{ie}^{e}\times \right)\tilde{C_{s}^{e}} & 0_{3\times 6} & \left(-\omega_{is}^{s}\times \right) \end{array}\right]$$

According to the new observation matrix ${\bar{H}}_{lg}$, we can see that its third element is still associated with the global variable $\tilde{C_{s}^{e}}$. From (55), we can see that the third column vector is determined by the error $dp_{l}^{e}$, whereas in the initial alignment phase the exact reference position $dp_{l}^{e}$ is small and therefore the third column vector is also small. ${\bar{H}}_{lg}$ can be treated as globally independent, so we establish the new observation matrix as satisfying the group affine property of ‘State–Trajectory Independence’.

### 3.3. Initialization of Alignment Parameters

Typically, the setting of the initial covariance matrix $P_{0}$ can seriously affect the filtering performance. Because the present method uses backward alignment, which only needs to be set before forward alignment, ref. [22] can be referred to, to set up $P_{l,0}$, which under the left-invariant error model is given as follows:

(56) $$P_{l,0}=T_{l}P_{0}T_{l}^{T}$$

(57) $$\mathrm{where}\;T_{l}=\left[\begin{array}{ccc} I_{3} & 0_{3} & 0_{3}\\ 0_{3} & -{\tilde{C}}_{e,0}^{sT} & -{\tilde{C}}_{e,0}^{sT}\left(\omega_{ie}^{e}\times \right)\\ 0_{3} & 0_{3} & -{\tilde{C}}_{e,0}^{sT} \end{array}\right]$$

### 3.4. Real-Time Performance

**Figure 2:**
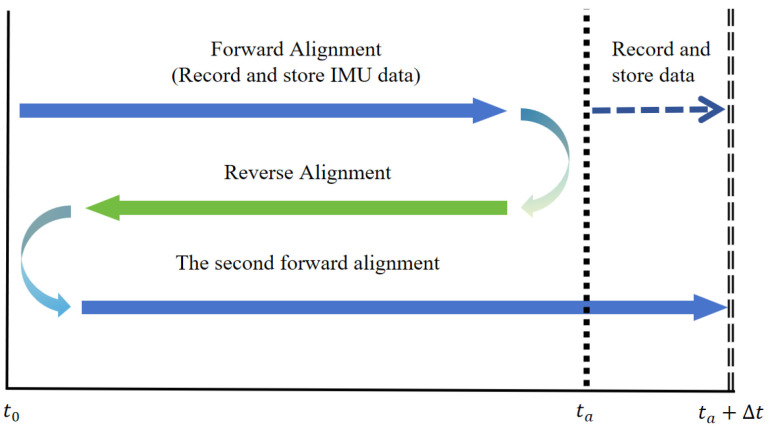
Inverse alignment diagram.

In this paper, the inverse alignment using the scheme in Figure 1 is for the sake of simplicity in principle explanation and consistency in experimental comparison. However, in practical applications, this method can perform real-time alignment, as shown in Figure 2. At time $t_{a}$ (the finish time in Figure 1), navigation will continue and record IMU data to facilitate a second forward alignment that catches up with the real-time moment. The time required for backtracking alignment, Δt, is very short; typically, with the efficient processing capabilities of computers, several minutes of alignment data processing can be completed in just a few seconds, ensuring the real-time performance of backtracking alignment.

## 4. Inverse Alignment and Navigation Tests

In order to verify the excellent performance of the proposed method for initial alignment on a real inertial guidance system, we conduct initial alignment and in-vehicle navigation experiments.

### 4.1. Alignment Accuracy Test

The following is an introduction of some instruments and basic parameters needed for the experiments.

**Figure 3:**
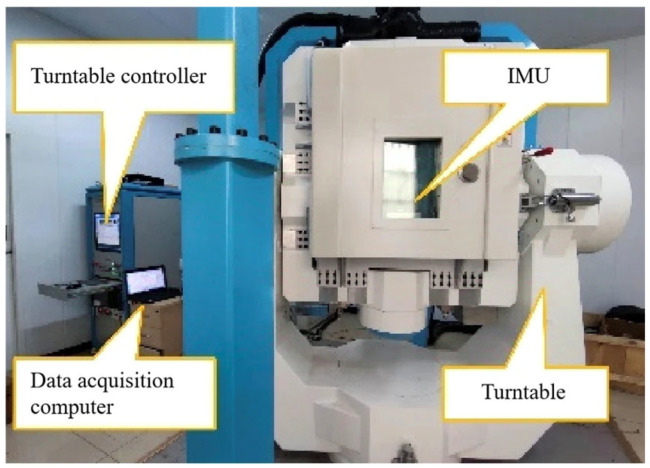
High-precision turntable.

Figure 3 shows an ultra-high-precision turntable with a measurement accuracy higher than 0.001°.

**Figure 4:**
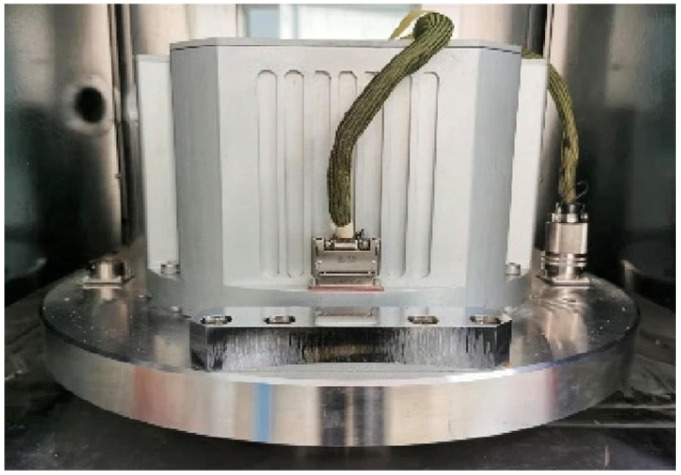
RLG IMU.

Figure 4 shows the ring laser gyro inertial measurement unit (IMU), which is placed in the inner frame of the ultra-high-precision turntable during the experiment.

Table 1 shows the specific parameters of the IMU used for the experiment.

Due to the limitations on the ultimate yaw accuracy of the initial alignment [28], we only analyze and verify the experimental results of the yaw angle alignment.

The alignment method employed in this paper is the RINS two-position alignment. This approach enables the zero bias of the gyroscope to be observable. Furthermore, based on the characteristics of inertial devices, yaw errors resulting from gyro drift and accelerometer bias can be effectively canceled after a certain rotation of the IMU.

After 12 consecutive hours of measurements, we monitored the yaw drift of the IMU (using the 15-state integrated navigation and drift estimation method). During the last 60 min of the measurement, we executed four 90° rotations of the IMU’s internal coordinate system. Subsequently, the yaw data of the IMU were recorded at different positions of the carousel (0°, 90°, 180°, and 270°). Through several independent drift measurement experiments, we obtained multiple sets of yaw data. Ultimately, we adopted the data in Table 2 as the baseline reference for yaw.

The initial alignment experiment was based on the benchmark shown in Table 2, and the rotating inertial navigation system performed multiple alignment tests at four positions and compared the alignment results. The alignment experiment was designed with three sets of scenarios as follows.

The first was the traditional initial alignment scheme (inertial coarse alignment and Kalman fine alignment), in which the coarse alignment time was 120 s and the fine alignment time was 180 s, a total of 300 s. The second scheme is the ordinary group affine alignment, and the alignment time is 300 s. The third is the inverse alignment scheme based on group affine proposed in this paper, with an alignment time of 300 s. In Table 3, the results of the alignment experiments with different schemes are compared.

In this experiment, we compared three different schemes: the traditional alignment method (inertial method and KF), the ordinary group radiation alignment, and the group affine inverse alignment method. Two replicate experiments were carried out under the same conditions and the same alignment time to ensure the reliability and accuracy of the experimental results.

In order to further verify the advantages of the alignment method proposed herein, we designed experiments to verify the fast performance of the alignment method.

In this experiment, the alignment time required for different schemes was compared with the same alignment accuracy. In order to ensure the consistency between the experiment and the previous experiments, the time allocation ratio of coarse alignment to fine alignment remained 2:3, the second group was the ordinary group affine alignment, and the third group was the group affine inverse alignment method proposed in this paper. In order to avoid experimental chance, we chose two different points for the comparison test. The experimental results are shown in Table 4.

**Figure 5:**
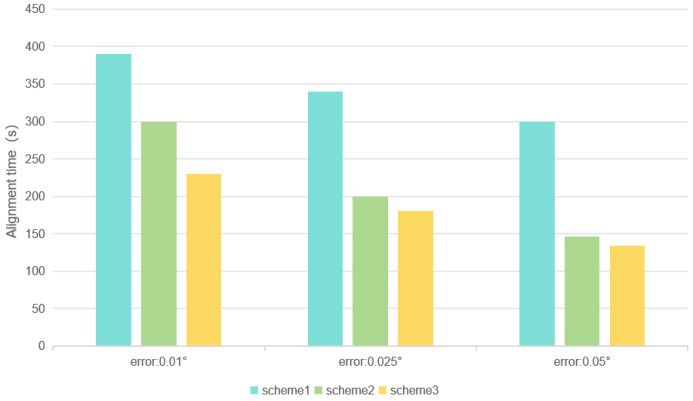
Alignment time for different scenarios.

In order to make the experimental data more obvious, we plotted the max of the experimental data, as shown in Figure 5. The vertical axis represents the alignment time, and the horizontal axis represents the alignment accuracy.

From the first experimental results, it can be concluded that the traditional alignment method (inertial method and KF) shows the basic alignment ability in the experiment, but its alignment accuracy is low and cannot satisfy the demands of high precision and fast start. Although the accuracy of the ordinary group radiation alignment method is improved compared to the traditional alignment method, it still cannot achieve the optimal balance between alignment time and alignment accuracy. The second experimental result shows the method that we proposed has a significant reduction in the alignment time required for the same alignment accuracy compared to the other two methods. The first experiment proved that the group affine inverse alignment method proposed in this paper achieves the highest accuracy of alignment in experiments, which proves the high efficiency of the method. A second experiment shows that this method has advantages in terms of alignment speed that cannot be matched by other alignment methods.

### 4.2. In-Vehicle Real-Time Navigation Test

**Figure 6:**
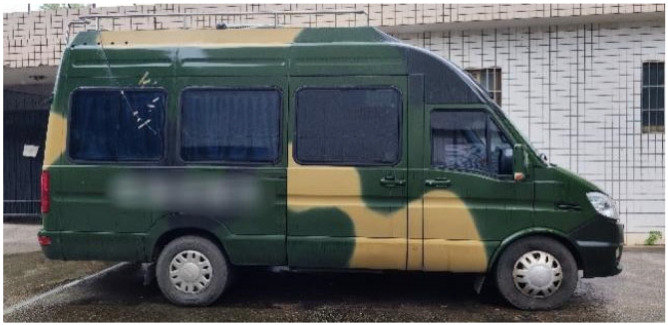
On-board experimental vehicle.

In order to further carry out the verification of the superiority of the initial alignment method proposed in this paper, we also conducted a real in-vehicle navigation experiment. The in-vehicle experiments verified the reliability and efficiency of the proposed method in real applications. Figure 6 shows the vehicle used for the on-board experiment.

**Figure 7:**
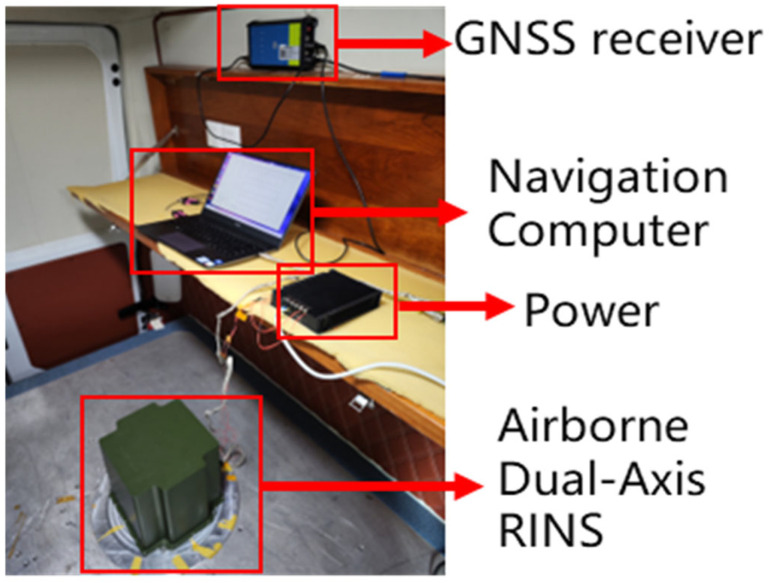
Rotating inertial navigation equipment.

Figure 7 shows the biaxial rotating inertial navigation system used in the experiment. The GNSS positioning system used in the experiment has an accuracy of less than 10 m, and the position information provided by GNSS is used as the reference position.

**Figure 8:**
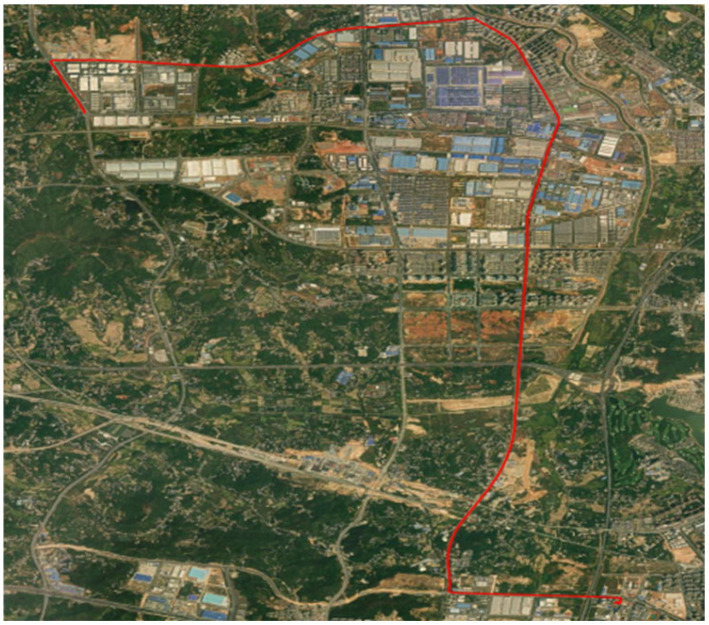
Route maps traveled by vehicles.

Figure 8 shows the route map of the vehicle used for on-board testing. The red line represents the driving trajectory.

In the experiment, the biaxial rotating inertial navigation device was turned on and held still for 200 s, and then its internal frame was rotated 180° and held still again for 200 s. After that, the dual-axis rotating inertial navigation system begins rotational modulation and performs an inertial navigation mission for up to 2.5 h.

Three schemes will be used to compare the alignment results: scheme 1 is the traditional alignment scheme (inertial method and KF, coarse alignment 120 s and fine alignment 180 s), scheme 2 is the ordinary group affine alignment scheme (the LSEGAKF method), and scheme 3 is the group affine inverse alignment scheme proposed in this paper, each with an initial alignment time of 300 s.

The results of the navigation experiments for the different schemes are shown below, in which the unit “nm” in the chart represents nautical miles.

**Figure 9:**
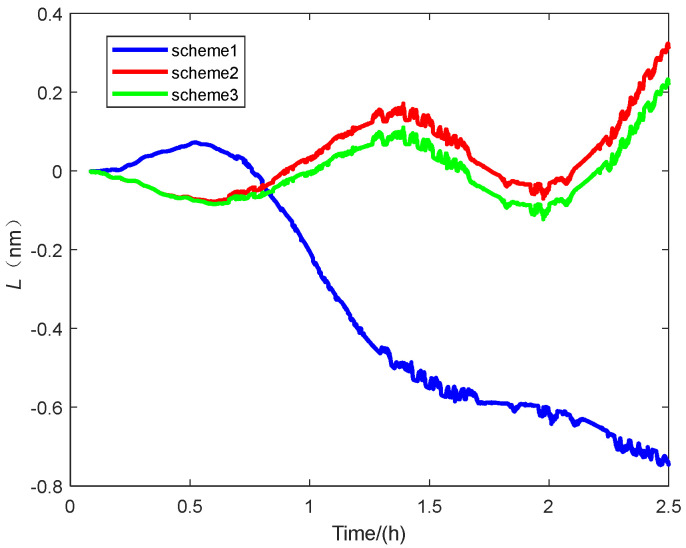
The latitude error.

Figure 9 shows the latitude error of the navigation experiment. The vertical axis represents the latitude error, and the horizontal axis represents time.

**Figure 10:**
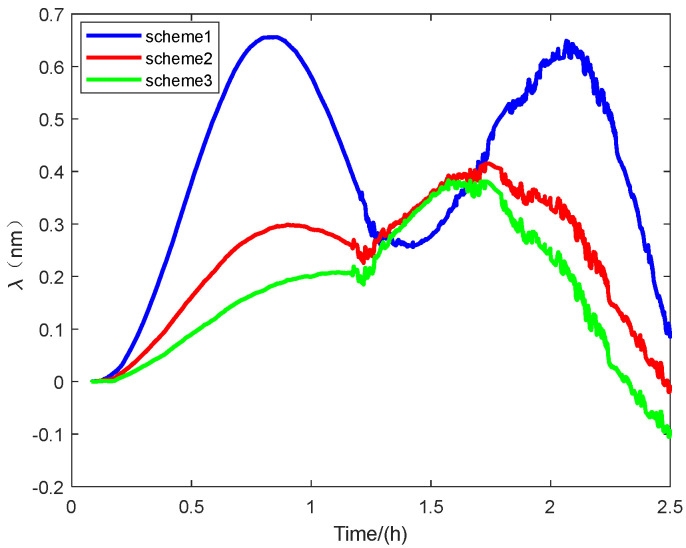
The Longitude error.

Figure 10 shows the longitude error of the navigation experiment. The vertical axis represents the longitude error, and the horizontal axis represents time.

**Figure 11:**
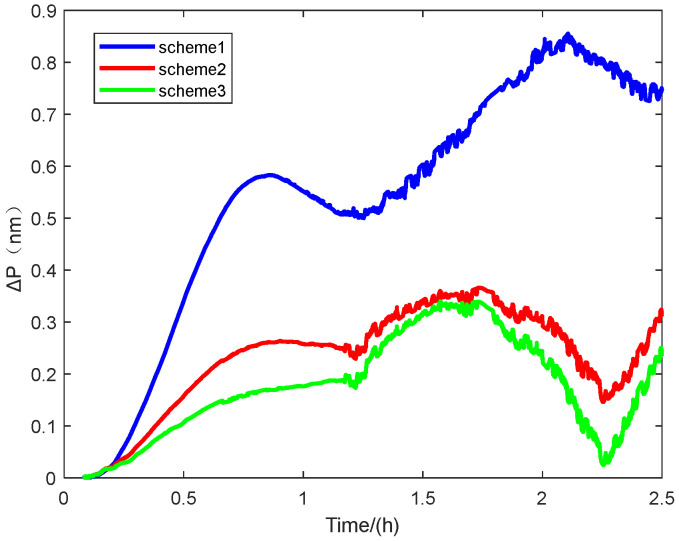
The positioning error.

Figure 11 shows the positioning error of the navigation experiment. The vertical axis represents the radial error of the navigation positioning, and the horizontal axis represents time.

As can be seen from the experimental results shown in Figure 8: Latitude error, Figure 9: Accuracy error, and Figure 11: Radial positioning error, this experiment completely simulates the real application scenario. The initial alignment was first carried out for 300 s, and then following the end of the direct in-vehicle navigation and localization, and the results show that the scheme 3 navigation and localization in this paper has the smallest error and the best performance.

Some specific parameters of the localization errors in this navigation experiment are also statistically presented to illustrate the superior performance of our proposed method in terms of initial alignment, as shown in Table 5.

The experimental results demonstrate that the proposed method achieves the smallest navigation error over 2.5 h. It enhances navigation accuracy by 60% compared to the traditional alignment method, which combines an inertial approach for coarse alignment and Kalman filtering for fine alignment. Furthermore, it improves accuracy by 7.2% compared to the common group affine alignment method. The 50% CEP of the navigation error is also reduced by 60% compared to conventional alignment methods and by 20% compared to normal group affine rapid alignment.

## 5. Conclusions

In this paper, we draw on Lie group and inertial navigation theory to incorporate the group affine property into the error model of a rotating inertial navigation system (RINS). Guided by the back-alignment concept, we propose a novel method that discards the traditional two-stage (coarse–fine) alignment approach in favor of an enhanced group affine alignment framework. This results in significant improvements in initial alignment performance. Specifically, we develop a novel model for inverse navigation and alignment rooted in the forward navigation and alignment theory of the RINS, incorporating the group affine property. We then combine forward and inverse alignments to create an innovative alignment technology. Experiments confirm that our method surpasses existing approaches in both required alignment time and accuracy, even with different misalignment angles, demonstrating its feasibility, novelty, and superiority. We plan to further explore moving-base or in-motion alignment schemes for rotating inertial navigation systems in follow-up research, extending the static-base alignment approach proposed in this paper to a broader range of application environments to meet practical demands for rapid startup, fast alignment, and high-precision alignment.

## Figures and Tables

**Table 1:** Parameters of IMU.

Error Type	Gyroscope	Accelerometer
Bias error	0.002°/h	20 μg
Random noise error	0.0003${}^{\circ}/\sqrt{h}$	20 μg/$\sqrt{Hz}$

**Table 2:** Yaw reference for different turntable positions.

Position	Reference (°)
1	−0.626
2	89.372
3	179.372
4	−90.626

**Table 3:** Alignment experiment results.

Scheme	Reference (°)	Result 1 (°)	Error 1 (°)	Result 2 (°)	Error 2 (°)
1	−90.626°	−90.653	0.027	−90.677	0.051
2		−90.624	−0.002	−90.620	−0.006
3		−90.627	0.001	−90.624	−0.002
1	89.372°	89.322	0.050	89.400	−0.028
2		89.359	0.013	89.351	0.021
3		89.378	−0.006	89.377	−0.005
1	179.372**°**	179.338	0.034	179.325	0.047
2		179.370	0.002	179.371	0.001
3		179.370	0.002	179.372	0.000
1	−0.626**°**	−0.651	0.025	−0.630	0.004
2		−0.628	0.002	−0.628	0.002
3		−0.625	−0.001	−0.625	−0.001

**Table 4:** Alignment times for different methods with the same heading angle error.

	Alignment Time (s)			
Error (°)		0.05	0.025	0.01
Point 1	Scheme 1	300	340	390
	Scheme 2	146	200	300
	Scheme 3	134	180	230
Point 2	Scheme 1	275	300	340
	Scheme 2	120	160	200
	Scheme 3	98	130	180

**Table 5:** Navigation positioning errors for different alignment schemes.

Scheme	Maximum of 2.5-h Navigation Error (nm)	50% CEP (nm)
1	0.8552	0.4767
2	0.366	0.2024
3	0.3397	0.1613

nm = nautical mile.

## Data Availability

Due to the sensitive nature of the data, we are unable to make them publicly available. However, reasonable requests for information regarding the data used in this study can be directed to the authors.
